# *Streptomycetaceae* and *Promicromonosporaceae*: Two Actinomycetes Families from Moroccan Oat Soils Enhancing Solubilization of Natural Phosphate

**DOI:** 10.3390/microorganisms10061116

**Published:** 2022-05-28

**Authors:** Meriam Bousselham, Sanaa Lemriss, Driss Dhiba, Yassine Aallam, Amal Souiri, Younes Abbas, Nezha Saïdi, Hassan Boukcim, Hanane Hamdali

**Affiliations:** 1 Laboratory of Agro-Industrial and Medical Biotechnologies, Faculty of Sciences and Technology, University of Sultan Moulay Slimane, Mghila BP 523, Beni-Mellal 23000, Morocco; bousselhameriam@gmail.com (M.B.); slemriss@lram-fgr.ma (S.L.); yassine.aallam@gmail.com (Y.A.); asouiri@lram-fgr.ma (A.S.); 2 Laboratory of Research and Medical Analysis of Gendarmerie Royale, Department of Biosafety PCL3, Rabat 10090, Morocco; 3 International Water Research Institute, University Mohammed 6 Polytechnic (UM6P), Hay Moulay Rachid, Ben Guerir 43150, Morocco; driss.dhiba@um6p.ma; 4 Polydisciplinary Faculty of Beni Mellal, University of Sultan Moulay Slimane, Mghila BP 523, Beni-Mellal 23000, Morocco; abbayouns@gmail.com; 5 CRRA Rabat, Plant Breeding, Conservation and Valorization of Plant Genetic Resources Research Unit, Rabat Institutes, P.O. Box 6570, Rabat 10101, Morocco; nezsaidi@yahoo.fr; 6 Scientific Parc Agropolis, 1900 la Lironde, PSIII Boulevard, F34980 Montferrier sur Lez, France; hassan.boukcim@valorhiz.com

**Keywords:** *Streptomyces*, *Promicromonospora*, biosolubilization, biofertilizers, rock phosphate, Moroccan oat soils

## Abstract

Soil actinomycetes explorations appear to be an efficient alternative as biofertilizers to optimize the use of phosphorus (P) resources and enhance plant growth. This research aimed to explore the distribution of actinomycetes isolated from four different rhizospheric Moroccan oat soils and to investigate their potential for P solubilization. The distribution of actinomycetes was significantly more abundant in Settat (9.68%), Tangier (7.38%), and Beni Mellal (6.87%) than in the Merchouch-Rabat (4.90%) region. A total of 235 actinomycete strains were isolated from all sites and tested for their ability to grow on a synthetic minimum medium (SMM) containing insoluble natural rock phosphate (RP) or synthetic tricalcium phosphate (TCP) as the unique P source. One hundred forty-three isolates (60.8%) had the ability to grow in the SMM with RP whereas only twenty-five isolates (17%) had the most active growth using the SMM with TCP. Eight isolates with the most active growth in solid SMM were selected for their P solubilization abilities in liquid SMM cultures. The highest amount of P solubilized was 163.8 µg/mL for RP and 110.27 µg/mL for TCP after 5 days of culture. The biosolubilization process of AM2, the most efficient RP and TCP solubilizing strain, probably implied the highest excretion of siderophore substances. Eight of these strains were shown to belong to the *Streptomyces* genus and one to the *Promicromonospora* genus. These findings bolster the phosphate biosolubilization abilities of actinomycetes and may participate in increasing agricultural yields in an eco-efficient and environmentally friendly manner.

## 1. Introduction

The growth of the world’s population over the last few decades is increasing. The current world population is 7.2 billion people; according to a United Nations report, this number is estimated to reach 9 billion in 2050 [1]. This demographic growth will increase demand for plant products and require the development of better and more innovative methods of plant production to meet the challenge of food security.

Oat (*Avena sativa* L.) is considered as one of the most important cereal crops for human consumption; it ranks sixth in world cereal production after wheat, maize, rice, barley, and sorghum [2]. In Morocco, oat production has increased from 14 kt in 1961 to 21 kt in 2018 [1] and oat has been classified as the third most-used fodder in Morocco, following alfalfa and barley [3]. Oat seeds are used for human consumption and gained considerable attention, especially for their nutritional value and health benefits [4], which is why the use of oat as animal feed has steadily declined. The properties of oats are known; it is an excellent source of starch (44.83–63.79%), carbohydrate (42.08–62.65%), protein (9.67–17.44%), fat (3.06–10.96%), β-glucan (1.37–6.05%), and ash (1.22–5.38%) [5,6]. Moreover, oats have different biological activities, such as wound healing, immune-modulatory, anti-diabetic, and anti-cholesterolemic effects [7], and they can be used in preparing functional foods that support a healthy lifestyle [8].

Oats, like other plants, need phosphorus (P) for growth; P is considered one of the main three nutrients most commonly found in crop fertilizers [9,10] and plays an important role in all metabolic processes, including photosynthesis, energy transfer, macromolecule biosynthesis, and respiration [11]. Furthermore, it is involved in the development of a good root system [12].

In soils, P can be found either in its organic or inorganic forms, but the abundance of this element may be a limiting factor for plant growth. The total soil P content is about 0.5%, but only 0.1% is available as a soluble form for plant uptake. This is due to its complex combination with other soil mineral contents [11,13]. For optimal productivity, the plant uses approximately 30 µmoL/L of the applied P chemical fertilizers [14]. The excessive use of chemical fertilizers may result in a potential negative impact on the environment, such as ground water, and soil degradation [15].

In modern agriculture, scientists are exploring the potential ability of microorganisms to colonize the soil, improve plant growth and development, and increase agricultural yield while respecting the environment [16,17]. In this regard, several studies have explored the beneficial effect of microorganisms for the solubilization of P to overcome the bioavailability constraints of this element for plants [11,18,19,20,21,22,23,24,25,26,27]. Actinomycetes are an example of these microorganisms [18,20,22,24,28,29,30,31,32,33,34]. They are the most ubiquitous group of bacteria in nature [35]. They exhibit a high diversity in genetic and functional variability in terms of their morphology, physiology, and metabolic capabilities. Ait Barka et al. [36] reported that actinobacteria is one of the most diverse taxonomic groups among the 18 major bacteria lineages currently identified, with 5 subclasses, 6 orders, and 14 suborders. Moreover, actinobacteria are known to be producers of a wide range of plant growth promoters and regulators, such as indole-3-acetic acid (IAA), cytokines, ACC deaminase, and various hydrolytic enzymes (cellulase, protease, keratinase, amylase, xylanase, lipase, and chitinase) [37]. All these value-added benefits in agriculture lead researchers to discover the great potential of these bacteria, notably in improving plant growth and as biocontrol agents [19,29,32,38]. However, the distribution of actinomycetes in Morocco and their use as biofertilizers for oat nutrition has not been yet explored. The aims of this work are to (i) explore the distribution of actinomycetes in oat rhizospheric soils of four different regions in Morocco, and (ii) evaluate actinomycetes’ contribution to the promotion of oat crop production.

## 2. Material and Methods

### 2.1. Study Area and Soil Sampling

**Figure 1:**
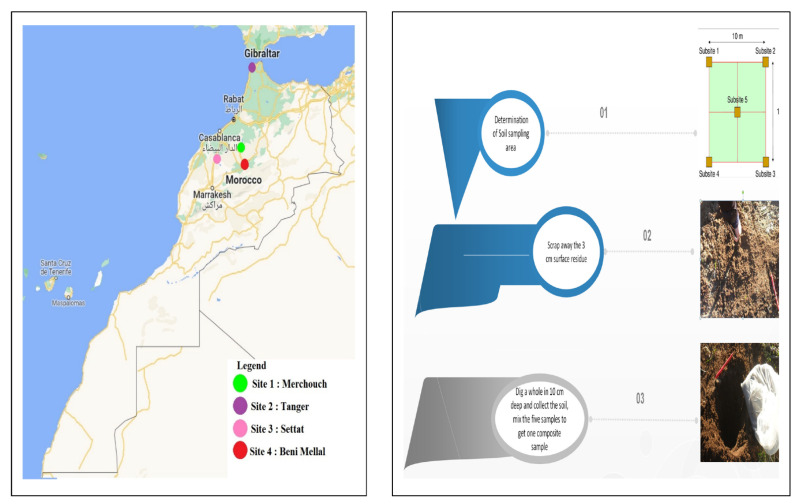
Sampling sites and schematic of the oat agricultural soils studied in Morocco.

The soil samples were collected in November 2018 from four different regions known as the most productive agricultural soils of oat in Morocco: Settat (33°7′16″ N; 7°37′48″ W), Tangier (35°43′48.6″ N; 5°52′57.2″ W), Rabat (33°60′499″ N; 6°71′600″ W), and Beni Mellal (32°19′26.144″ N; 6°22′50.988″ W) (Figure 1).

From each sampling point, 3 cm surface residues were first removed and five subsamples, a distance of 10 m from each other in different directions, were collected from 0 to 10 cm depth and thoroughly mixed to ensure sample homogeneity. All soil samples were then air-dried, homogenized, sieved (<2 mm), placed in a sterile tightly closed polyethylene bag, and stored at 4 °C.

### 2.2. Physicochemical Analysis

pH and electrical conductivity (EC) measurements were performed in a soil–water suspension using a pH meter (PHSJ-3F) and conductivity meter (DDS-12DW), respectively. Organic matter (OM%) was deduced from the organic carbon percentage using Anne’s method, which is based on potassium dichromate (K_2_Cr_2_O_7_) oxidation of the organic fraction present in the sample [39]. Kjeldahl’s method was used to obtain total Kjeldahl’s nitrogen (TKN). Phosphorus content was determined using the method described by Olsen and Sommers [40]. Soil particle size was determined using a pipetting method (United State Salinity Laboratory Staff, 1954, Washington, DC, USA).

### 2.3. Isolation of Total Flora and Actinomycetes from Oat Soils

First, 2 g (fresh weight) of each soil sample were resuspended in 18 mL of sterile physiological serum (9 g/L, NaCl), homogenized and sonicated according to Qin et al. [41]. Then, 0.1 mL of various dilutions of the treated samples was plated in triplicate on the surface of nutrient agar (Difco, Sparks, MD, USA) for Gram-positive and -negative bacteria and of synthetic minimum medium (SMM) containing 10 g/L glucose, 2 g/L NaNO_3_, 0.5 g/L MgSO_4_·7H_2_O, 0.5 g/L KCl, 0.01 g/L FeSO_4_·7H_2_O, and K_2_HPO_4_ (0.5 g/L, 4.38 mM) as described previously by Hamdali et al. [22]. The pH of these media was adjusted to 7 and they were sterilized at 121 °C for 20 min. These media were supplemented with 40 mg/mL of actidione and 10 mg/mL of nalidixic acid to inhibit growth of fungi and Gram-negative bacteria, respectively. After plating, the agar plates were incubated for 21 days at 28 °C in order to allow growth of the slow-growing actinomycetes. They were recognized according to morphological features following the International Streptomyces Project (ISP) [42].

### 2.4. Screening for Actinomycetes Able to Use Rock Phosphate (RP) and Tricalcium Phosphate (TCP) as Unique P Source

The selection of actinomycetes able to use RP originating from Khouribga phosphate mine in Morocco, as the sole P source, was carried out by plating isolated colonies on the SMM containing 0.5 g/L of RP (approximately equivalent to 2.2 mM phosphorus) as a unique P source or on the SMM containing soluble K_2_HPO_4_ (0.5 g/L 4.38 mM) or no P source. Spores of actinomycete strains showing the most active growth on SMM containing RP as sole P source were stored in 20% (*w*/*v*) sterile glycerol at −20 °C and were subsequently tested for their ability to grow on SMM containing TCP (0.5 g/L, Ca_3_(PO_4_)_2_) (Sigma Aldrich, Darmstadt, France) as a unique P source.

### 2.5. Quantitative Estimation of the Amount Soluble P Released in the Growth Medium by the Selected Actinomycete Strains

The selected actinomycete strains were inoculated at 10^6^ spores/mL in 250 mL Erlenmeyer flasks containing 50 mL of liquid SMM medium with 0.5 g/L RP or 0.5 g/L TCP as the sole P source, in triplicate, and grown for 5 days at 28 °C on a rotary shaker (180 g/min) [31]. Every day, 1 mL aliquot of each culture was taken and centrifuged at 10,000× *g* for 10 min, and the pH of the supernatant was determined. The supernatant was analyzed for P_2_O_5_ content by the chlorostannous reduced molybdophosphoric acid blue colorimetric method [40]. Similar measures were carried out in non-inoculated flasks incubated in the same conditions to determine the amount of free available phosphate released from RP and TCP.

### 2.6. Siderophore Production Test: CAS Agar Plate Technique

Siderophore production of the most efficient P solubilizing strains was determined using Chrome Azurol S (CAS) agar plate method described by Schwyn and Neilands [43]. Agar plugs (10 mm diameter) of actinomycete strains grown on solid SMM medium containing 0.5 g/L TCP as the sole P source were spotted separately on CAS medium and incubated for 3 days at 28°. After incubation, the apparition of a yellow halo around the plugs indicated the production of siderophores.

### 2.7. Morphological, Physiological, and Chemotaxonomic Characterization of the Selected Strains

The morphological, cultural, physiological, and biochemical characteristics of the selected strains were evaluated as described in the International Streptomyces Project [42]. The assimilation of carbohydrates was determined using ISP9 medium containing 10 different carbohydrates, as the sole carbon source, at a concentration of 1% (*w*/*v*).

### 2.8. Amplification and Sequencing of the 16S rDNA of the Selected Strains

Genomic DNA was isolated from pure cultures in Bennet agar medium at 37 °C of eight selected strains (AM1, AM2, AM3, AM5, AM6, AM13, AM15, AM24) and was extracted using the Maxwell^®^ RSC Instrument (Promega, Madison, WI, USA) and the Maxwell^®^ RSC PureFood GMO and Authentication Kit (Promega) according to the manufacturer’s recommended protocol. The PCR mixture, with a total volume of 25 μL, was prepared using the AccuPower Taq PCR PreMix (Bioneer, Oakland, CA, USA) and 16S rRNA gene primers: 27fM (5′-AGAGTTTGATCMTGGCTCAG-3′) and 1492rY (5′-GYTACCTTGTTACGACTT-3′) [44]. PCR conditions were as follows: after initial denaturation (96 °C for 1 min), 30 cycles of 96 °C for 30 s, 60 °C for 30 s and 72 °C for 1 min 30 s were performed, followed by a final extension (5 min, 72 °C). Amplification was carried out using a GeneAmp PCR 9700 System (Applied Biosystems). Negative controls were included with no addition of template DNA. PCR products visualized on a 2% (*w*/*v*) agarose gel stained with ethidium bromide were sequenced bidirectionally with 27F and 1492R primers using a Sanger sequencer.

Sequences similarities were performed using the online sequence analysis resources LEBIBI database [45] and GenBank through Nucleotide BLAST (http://www.ncbi.nlm.nih.gov/BLAST/ accessed on 8 April 2021, for AM2, AM5, AM6 and 27 May 2021 for AM13, AM15, and 14 October 2021, for AM1, AM3, AM24). Unrooted phylogenetic trees were inferred using the neighbor-joining method [46]. The percentage of replicate tree in which the associated taxa clustered together in the bootstrap test (1000 replicates) is shown next to the branches [47]. The evolutionary distances were computed using the Kimura 2-parameter method [48] and are in the units of the number of base substitutions per site. Evolutionary analyses were conducted in MEGA X [49].

### 2.9. Statistical Analysis

Statistical analysis of soil chemical parameters, total flora, and actinomycete strains distribution was performed using ANOVA. The Duncan test was used to compare the average abundance and percentage contribution of the actinomycete isolates to the total flora in the four studied sites. All values are the means of three replicates plates from the same soil sample. Least significant difference (LSD) was used to compare the parameter concentrations between sampling sites and standard deviation was calculated using SPSS statistical software, version 23.0 (IBM, New York, NY, USA).

## 3. Results

### 3.1. Soil Properties and Distribution of Actinomycetes

To elucidate relationships among actinomycetes abundance and soil physicochemical properties, we compared their densities from the four different oat rhizospheric soils. Highly significant (*p* < 0.001) differences mainly in the percentage of actinomycetes, the conductivity, and the granulometry parameters (fine silt, large silt, fine sand, large sand, and clay) were observed (Table S1). The conductivity revealed that all the soils tested were non-saline (Table S1). P content was high in site 3 (0.84 mg/L) in comparison to sites 2, 4, and 1 (0.57, 0.37, and 0.27 mg/L, respectively). The granulometry shows that site 1 contains a large amount of clay (89.29%) compared with the other sites. Interestingly, we observed that sites 2 and 3 contained more actinomycetes than sites 1 and 4 (Table S1). This might be due to the geographical position characterized by typical soil (rich in organic matter and P) and climate properties.

### 3.2. Isolation of Actinomycetes from Four Different Sites

The distribution of total flora (TF) and actinomycetes of oat rhizospheric soils collected from four sites was evaluated in this study. The actinomycete strains were significantly more abundant in site 2 (7.38%), site 3 (9.68%), and site 4 (6.87%) than in site 1 (4.90%).

From a total of 235 isolates, only 143 strains (60.8%) could use RP when plated on solid SMM + RP as the unique P source, suggesting that they were able to use the P trapped in the RP for their own growth. Among these 143, only 25 isolates (17%) had also the ability to grow on SMM + TCP as the sole P source. Eight of the twenty-five isolates with the highest solubilization rates on SMM + RP and SMM + TCP and characterized with different morphological traits were selected for more extensive studies. AM1 and AM13 were isolated from site 1; AM6 was isolated from site 2; AM2, AM3, and AM5 were isolated from site 3; and AM15 and AM24 were isolated from site 4.

### 3.3. Quantitative Estimation of the P Concentration Solubilized by the Selected Actinomycetes

**Figure 2:**
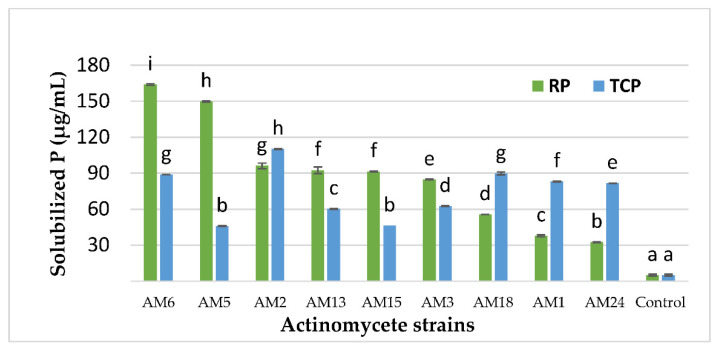
Concentration of soluble phosphate released (µg/mL) from tricalcium phosphate (TCP) and natural rock phosphate (RP) in the non-inoculated flasks (control) and in the supernatant cultures of the eight selected actinomycete isolates grown for five days in SMM containing 0.5 g/L RP or 0.5 g/L TCP. Error bars represent standard deviations of the mean values of the results of three independent culture replicates. Different lowercase letters above bars indicate significant differences between treatments at *p* ≤ 0.05.

The selected actinobacteria strains showed different abilities to release soluble P from RP (Figure 2). Phosphorus release ranged from 32.5 to 163.8 µg/mL. The AM2, AM5, and AM6 strains were the most efficient strains, releasing 96.11, 149.72 µg/mL, and 163.88 µg/mL of soluble P in the growth medium after 5 days of cultivation, respectively (Figure 2).

In order to confirm the P biosolubilization by the selected actinomycete strains, we tested them in the same conditions with the purified synthetic TCP as the sole P source. The results showed different effects of the selected actinomycete strains on TCP biosolubilization with different abilities to release soluble P (ranged from 45.83 to 110.28 µg/mL) from TCP and different actinomycete strain efficiency (Figure 2). AM2 and AM6 isolates were the most efficient strains, releasing 110.27 µg/mL and 88.89 µg/mL of soluble P in the growth medium after 5 days of cultivation, respectively (Figure 2). The solubilized amount of free available phosphate released from TCP in the non-inoculated flask (control) was 5 µg/mL.

### 3.4. Investigation of the P Biosolubilization Mechanism

The pH of the growth medium of the eight selected strains in RP and in TCP showed a slight alkalinization of the medium, ranging from 6.6 to 7.7 at the end of growth (Table 1). It is noteworthy to mention that AM6, AM5, AM2, and AM13 strains are the highest solubilizers of both RP and TCP, whereas AM1, AM3, AM15, and AM24 are the less efficient P solubilizing strains.

**Figure 3:**
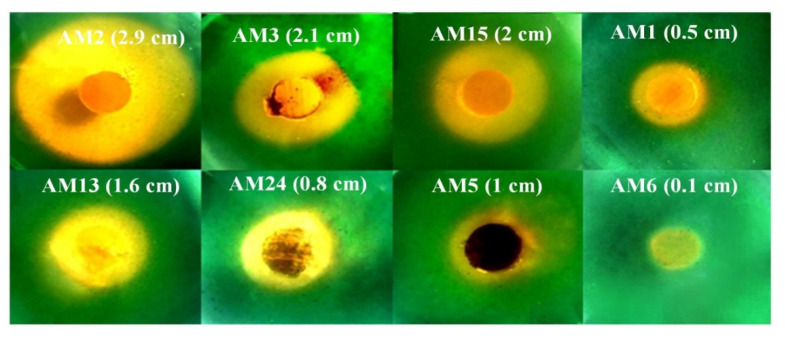
Halo of discoloration of CAS-agar around agar plugs of the eight most efficient TCP/RP solubilizing actinomycete isolates grown for 5 days in solid SMM containing 0.5 g/L TCP and deposited on the surface of a CAS–blue agar plate. Diameters of the halos are expressed in cm.

The CAS-agar test shows that all the eight TCP and RP solubilizing actinomycete strains produce siderophore substances (Figure 3). AM2, AM5, AM6, and AM13 are the most siderophore-producing, as judged by the size and the intensity of the color change of the CAS-agar, whereas the AM1, AM3, AM15, and AM24 strains secreted less of these substances (Figure 3).

### 3.5. Biochemical and Taxonomical Characterization of the Selected Strains

In order to determine whether the eight strains were similar or different, their ability to assimilate different carbon sources (eight sources) was tested. All strains were able to use mannitol, lactose, glucose, fructose, maltose, sorbitol, citrate, and starch as the sole carbon source (Table 2). Strains were also evaluated for their ability to withstand salt stress by growing them in NaCl concentrations of 5, 7.5, and 9.5 g/L. All strains showed the best growth at 9.5 g/L NaCl (Table 2), except AM15, which showed better growth at 7.5 g/L NaCl (Table 2). Therefore, these strains could potentially be halotolerant.

The eight sequences of the 16S rRNA gene were analyzed by comparison with LEBIBI database [45] and GenBank through Nucleotide BLAST (http://www.ncbi.nlm.nih.gov/BLAST/ (accessed on 3 April 2021)). Nucleotide sequences of partial 16S rRNA of the identified strains were deposited into the GenBank Database (http://www.ncbi.nlm.nih.gov/GenBank/ (accessed on 3 April 2021)) under the accession numbers listed in Table 3.

**Figure 4:**
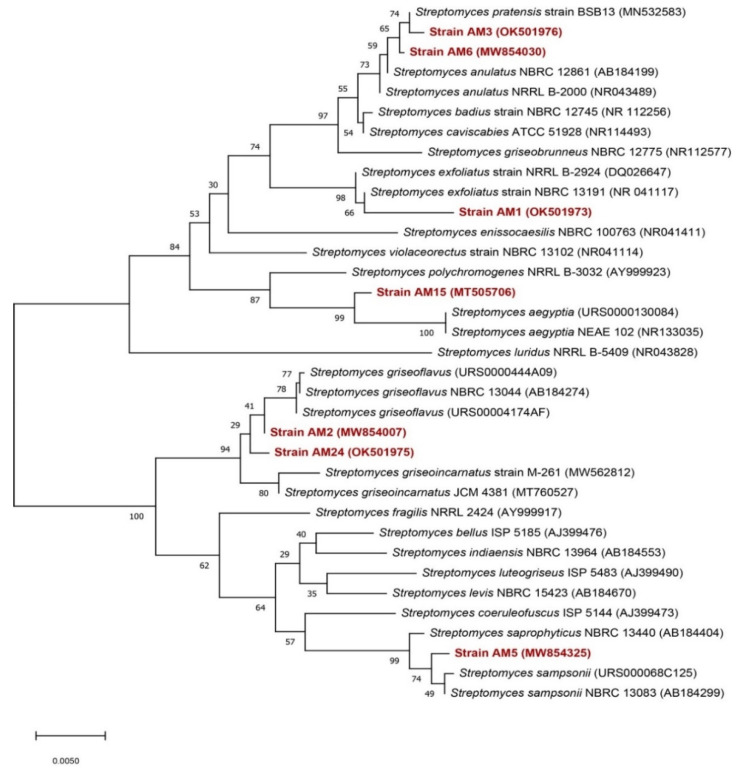
Neighbor-joining phylogenetic tree of the 7 isolated strains and 28 *Streptomyces* species based on nearly complete 16S rRNA gene sequences (1400 nt). Numbers at the nodes indicate levels of bootstrap support (%) based on a neighbor-joining analysis of 1000 resampled datasets; only values >50% are given. Accession numbers are given in parentheses.

**Figure 5:**
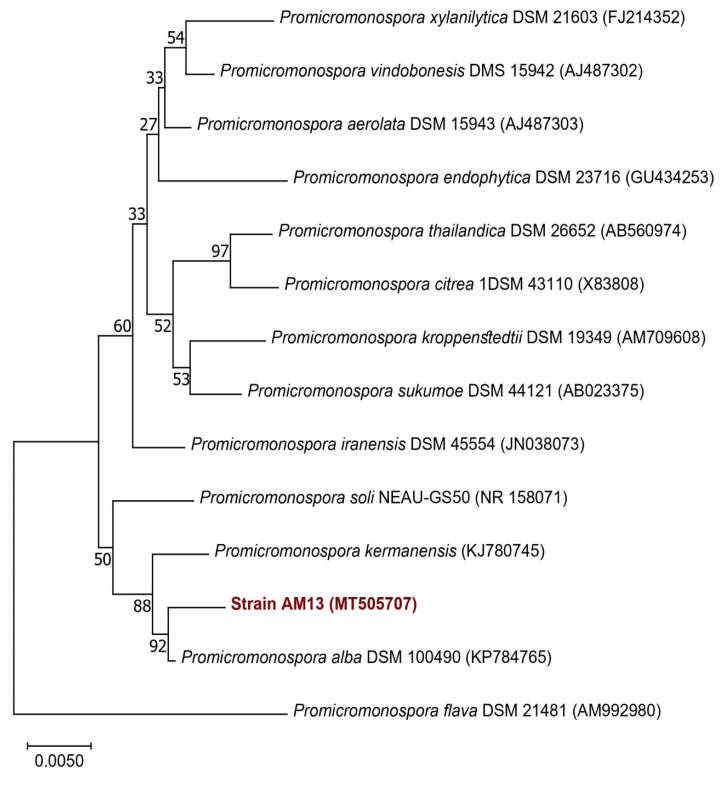
Neighbor-joining phylogenetic tree of the isolated actinomycete strain AM13 and 13 *Promicromonospora* species based on nearly complete 16S rRNA gene sequences (1400 nt). Numbers at nodes indicate levels of bootstrap support (%) based on a neighbor-joining analysis of 1000 resampled datasets; only values >50% are given.

Neighbor-joining trees based on 16S rDNA gene sequences were generated and show the positions of the studied strains and related strains (Figure 4 and Figure 5), using 1490 bp of aligned sequences and the closest matches to each isolate that were identified at the species level (Table 3). Twenty-eight *Streptomyces* species and thirteen *Promicromonospora* species were retrieved from GenBank and used in the construction of phylogenic trees (Figure 4 and Figure 5). Phylogenetic analysis revealed that the seven strains AM1, AM2, AM3, AM5, AM6, AM15, and AM24 belong to the *Streptomycetaceae* family and AM13 belongs to the *Promicromonosporaceae* family.

## 4. Discussion

Phosphorus-solubilizing microorganisms are microbial fertilizers with a wide range of potential applications [50,51]. Tahir et al. [52] found that inoculation with PSB significantly increased wheat growth rate and phosphorus uptake, and wheat yield increased by 10–12%.

To our knowledge, this is the first report on the diversity and the PGP abilities of endemic actinomycete strains isolated from oat agricultural soils in different Moroccan regions. Results revealed that actinomycete flora was different in terms of percentages and composition from site to site in the studied locations (Figure 5, Table S1), despite the geographical location, soil properties, or the climate characteristics in our study. Bhatti et al. [29], Alam et al. [53], and Hamdali et al. [31] reported that actinomycetes have a large capacity for the decomposition of complex organic matter via the excretion of numerous hydrolytic enzymes that allow them to thrive in a variety of ecological environments while enhancing plant nutrition and serving as biocontrol agents.

In this study, differences in soil properties and the percentage of actinomycetes were identified in the targeted soils. Samples containing the highest amounts of phosphorus and organic matter were soil from sites 2 and 3. These two soil types have the most abundant of actinomycetes flora, which indicates that the distribution of actinomycetes may be influenced by the soil properties and composition—mainly the phosphorus and organic matter contents. Several studies have shown that soil properties are more important for bacterial soil composition [54,55]. Among all soil properties, organic matter, salinity, relative moisture, temperature, pH, and vegetation are important factors that control the abundance of actinomycetes in a particular type of soil [56]. Our study revealed that the phosphorus content is one of the crucial factors impacting the actinomycetal communities. According to Seshachala and Tallapragada [57], the variation in actinomycetes abundance in the different soil ecosystems indicates that soils with higher organic content contained a large number and variety of rhizospheric soil bacteria and fungi capable of phosphate solubilization.

Of the 253 (60.8%) initial isolates, 143 were able to grow in SMM with RP; among these 143 strains, only 25 isolates (17%) showed the most active growth using the SMM with TCP. These results are different from those of Aallam et al. [18], who found that 28% of actinomycetes isolated from sugar beet soils had the ability to grow on RP and 47.36% also had the ability to grow on TCP as unique phosphate source.

All of the tested strains showed different abilities to solubilize TCP and RP and differences among the phosphate solubilized in the same strain. It reached up to 110.27 µg/mL for TCP and 163.8 µg/mL for RP. These amounts are significantly higher than the phosphate biosolubilization abilities of actinobacteria isolated from Moroccan phosphate mines [22] where the maximum amount is in the vicinity of 30 µg/mL of P released from RP. On the contrary, Aallam et al. [18] isolated actinomycete strains able to release up to 170 µg/mL from TCP and 150 µg/mL from RP, which is slightly higher than our findings. This difference in acquisition of phosphate from these two insoluble sources may be explained by the genomic diversity of actinomycetes that provides them with physiological, metabolic characteristics, and biochemical ability to produce and release the appropriate substances for biosolubilization of each strain isolated.

Tian et al. [58] reported that *Bacillus* sp., *Pseudomonas* sp., *Rhizobium* sp., and *Escherichia* sp. can release orthophosphate from tricalcium phosphate, apatite, fluorapatite, and phosphorites, mainly through acidolysis. In fact, Zhou et al. [59] reported that PSM *Pseudomonas fluorescens* can dissolve fluorapatite as its sole P source in acidified medium [60,61,62]. Otherwise, the P-solubilizing activity is determined by the acids, which through their carboxylic groups, chelate the cations (mainly Ca) bound to phosphate and convert them into soluble forms [63].

The current study demonstrated that the most effective RP and TCP solubilizing strains do not acidify the culture medium, which explains inadequate allocation of organic acids but better excretion of chelator substances as revealed by the blue CAS–agar test [43,64]. The strong electrostatic interactions between the constituents of the RP and the P would be destroyed by these chelators, destabilizing the RP structure and thus liberating P. The free negatively charged phosphates are thought to attract the protons of the growth medium when the RP elements are trapped by the chelator, reducing the concentration of free protons in the medium and thus accounting for the observed alkalinization of the growth medium [31]. These results confirmed other studies that strengthen the ability of actinomycete strains to solubilize P by the production of siderophores [23,24,31].

Our curiosity about the diversity of these actinomycetes has led us to demystify the type of bacteria in this microbial community. Indeed, the isolated actinomycete strains belong to both the families of *Streptomycetaceae* and *Promicromonosporaceae*. The species involved in this community are *Streptomyces griseoflavus*, *Streptomyces sampsonii*, *Streptomyces anulatus*, *Streptomyces aegyptia*, *Streptomyces exfoliates*, *Streptomyces pratensis*, *Streptomyces griseoincarnatus*, and *Promicromonospora alba*. These species have shown particularly attractive biochemical traits such as using mannitol, lactose, glucose, fructose, maltose, sorbitol, citrate, and starch as unique carbon sources. Furthermore, their potential to withstand salt stress is worth exploring to better elucidate the eco-efficiency of these actinomycetes. Recently, advanced research into coastal salt marsh rhizosphere soils showed that *Streptomyces* sp. promote the growth of wheat seedlings with and without NaCl [65]. This goes to say that these rhizosphere bacteria are producers of bioactive metabolites help to improve the soil fertility, promote plant growth and development, and provide defense against phytopathogens, and have the ability to withstand various environmental stresses.

## 5. Conclusions

This study highlights the importance of actinomycetes isolated from Moroccan oat rhizospheric soils. Results showed the widely distribution of actinomycetes in Settat and Tangier regions where rhizospheric soils exhibit a significant amount of P. Furthermore, actinobacteria are able to release soluble P from both forms of insoluble P (RP and TCP). The AM2, AM5, AM6, and AM13 strains exhibited greater P biosolubilization abilities. The ability of these strains to solubilize RP and TCP did not involve the excretion of organic acids but likely siderophore substances that are currently being purifieed and structurally analyzed. It was shown in our study that the isolated actinomycete strains belong to the families of *Streptomycetaceae* and *Promicromonosporaceae*, and the *Streptomyces* genus dominated. Further studies on P biosolubilization by the selected actinomycete strains in the presence of oat plants are under investigation. Thus, the use of RP as a natural P fertilizer with spores of AM2 could be considered as eco-friendly and sustainable bio-phosphate fertilizers to boost oat crop growth, development, and yield.

## Figures and Tables

**Table 1:** Evolution of the pH of the medium of the eight selected actinomycete isolates grown in SMM + TCP and SMM + RP. Standard deviations represent mean values of results from three independent culture replicates.

	pH									
	Day 1		Day 2		Day 3		Day 4		Day 5	
	RP	TCP	RP	TCP	RP	TCP	RP	TCP	RP	TCP
AM2	6.88 ± 0.01	6.55 ± 0.21	6.93 ± 0.04	6.75 ± 0.21	7.09 ± 0.01	7.05 ± 0.06	7.35 ± 0.07	7.24 ± 0.01	7.47 ± 0.04	7.60 ± 0.28
AM5	6.80 ± 0.01	6.80 ± 0.01	6.86 ± 0.01	6.93 ± 0.13	6.80 ± 0.00	7.16 ± 0.04	7.12 ± 0.04	7.5 ± 0.01	7.62 ± 0.04	7.55 ± 0.07
AM6	6.815 ± 0.01	6.81 ± 0.01	7.19 ± 0.01	7.22 ± 0.02	7.10 ± 0.13	7.25 ± 0.02	6.90 ± 0.00	7.46 ± 0.06	7.23 ± 0.01	7.52 ± 0.02
AM13	6.65 ± 0.07	6.82 ± 0.02	6.89 ± 0.01	6.90 ± 0.17	6.91 ± 0.01	7.06 ± 0.05	7.07 ± 0.01	7.10 ± 0.02	7.71 ± 0.01	7.59 ± 0.14
AM15	6.03 ± 0.02	6.82 ± 0.04	6.85 ± 0.07	6.80 ± 0.00	7.43 ± 0.04	7.46 ± 0.06	7.43 ± 0.11	7.45 ± 0.06	7.49 ± 0.04	7.5 ± 0.06
AM1	6.8 ± 0.01	6.75 ± 0.07	6.88 ± 0.02	6.8 ± 0.01	7.1 ± 0.02	7.23 ± 0.14	7.3 ± 0.01	7.7 ± 0.03	7.35 ± 0.01	7.81 ± 0.01
AM3	6 ± 0.01	6.9 ± 0.01	6.5 ± 0.05	7.08 ± 0.01	6.59 ± 0.00	7.43 ± 0.11	6.8 ± 0.02	7.6 ± 0.01	6.86 ± 0.14	7.8 ± 0.01
AM24	7.01 ± 0.01	6.5 ± 0.05	7.14 ± 0.01	6.67 ± 0.02	7.28 ± 0.13	7.12 ± 0.00	7.45 ± 0.04	7.32 ± 0.01	7.7 ± 0.01	7.56 ± 0.017

**Table 2:** Biochemical and morphological characteristics of the eight selected isolates.

Strains	AM2	AM5	AM6	AM13	AM15	AM1	AM3	AM24
Origin	Site 3		Site 2	Site 1	Site 4	Site 1	Site 3	Site 4
Aerial spore mass	Yellow	White	Gray	White	Pink	Yellow	White	Brown
Soluble pigment	-	Green	-	Beige	-	-	-	-
Colony reverse	Yellow	Brown	Brown	Beige	White	White	White	Brown
C. source utilization								
Glucose	+++	+++	+++	+++	+++	+++	+++	+++
Fructose	+++	+++	+++	+++	++	+	++	+
Maltose	+++	+++	+++	+++	++	++	+	+
Lactose	+++	+++	+++	+++	++	+	++	+
Amidon	+++	+++	+++	+++	++	+	+	+
Mannitol	+++	+++	+++	+++	++	+	+	++
Sorbitol	+++	+++	+++	+++	+	++	++	+
Citrate	+++	+++	+++	+++	+	+	+	+
Salinity resistance % (maximum of resistance to NaCl on g/L)	9.5	9.5	9.5	9.5	7.5	7.5	2.5	9.5
pH resistance	5–13	5–7	5–10	5–13	5–13	5–13	7–10	5–13

+++: High growth; ++: Medium growth; +: Low growth; -: Absence.

**Table 3:** 16S rRNA identification of the eight selected isolates.

Strains	16S rRNA Identification	Accession Number
AM2	*Streptomyces griseoflavus*	MW854007
AM5	*Streptomyces* *sampsonii*	MW854325
AM6	*Streptomyces anulatus*	MW854030
AM15	*Streptomyces aegyptia*	MT505706
AM13	*Promicromonospora alba*	MT505707
AM1	*Streptomyces exfoliates*	OK501973
AM3	*Streptomyces pratensis*	OK501976
AM24	*Streptomyces griseoincarnatus*	OK501975

## Data Availability

Not applicable.

## Supplementary Materials

The following supporting information can be downloaded at: https://www.mdpi.com/article/10.3390/microorganisms10061116/s1, Table S1: Physicochemical characteristics of the four oat agricultural soils of the different sites studied in Morocco. Different lowercase letters above numbers show significant differences between sites at *p* ≤ 0.05.
