# Supplementary material for: Streptomycetaceae and Promicromonosporaceae: Two Actinomycetes Families from Moroccan Oat Soils Enhancing Solubilization of Natural Phosphate
Source: Microorganisms. 2022 May 28;10(6):1116. doi: 10.3390/microorganisms10061116 (PMC9230749; doi:10.3390/microorganisms10061116)
Supplement: Supplementary file 1 [file microorganisms-10-01116-s001.zip › microorganisms-1697220-supplementary.pdf]

microorganisms MDPI

Supplementary materials

Table S1. Physicochemical characteristics of the four oat agricultural soils of the different sites studied in Morocco. Different lowercase letters above numbers show significant differences between sites at $p \leq 0.05$.

		Mean	Standard Deviation	ANOVA
pH	Site 1	8.04 b	0.04	
	Site 2	7.41 a	0.09	
	Site 3	7.74 b	0.20	
	Site 4	7.96 b	0.06	P<0.05
Conductivity (µS/m)	Site 1	113.90 a	5.80	
	Site 2	158.00 b	2.83	
	Site 3	152.60 b	0.57	
	Site 4	990.50 c	13.44	P<0.05
Organic matter (%)	Site 1	3.14 a	2.23	
	Site 2	3.69 a	0.000	
	Site 3	4.12 a	0.21	
	Site 4	3.42 a	0.2	Ns
Phosphorus (mg/L)	Site 1	0.27 a	0.01	
	Site 2	0.57 ab	0.07	
	Site 3	0.84 b	0.11	
	Site 4	0.37 a	0.15	P<0.05
Nitrogen (%)	Site 1	0.04a	0.00	
	Site 2	0.13a	0.17	
	Site 3	0.13a	0.17	
	Site 4	0.42a	0.32	Ns
Fine sand	Site 1	4.46 c	0.00	
	Site 2	20.76 d	0.00	
	Site 3	1.22 a	0.00	
	Site 4	1.91 b	0.00	P<0.05
Coarse sand	Site 1	1.78 c	0.00	
	Site 2	16.35 d	0.00	
	Site 3	0.25 a	0.00	
	Site 4	1.29 b	0.00	P<0.05
Fine silt	Site 1	4.47 a	0.00	
	Site 2	12.58 b	0.00	
	Site 3	32.93 d	0.00	
	Site 4	21.16 c	0.00	P<0.05
Coarse silt	Site 1	0.00 a	0.00	
	Site 2	17.62 b	0.00	
	Site 3	31.38 c	0.01	
	Site 4	41.43 d	0.00	P<0.05
Clay	Site 1	89.29 d	0.00	
	Site 2	32.71 a	0.00	
	Site 3	34.23 c	0.00	
	Site 4	34.22 b	0.00	P<0.05
Actinomycetes (%)	Site 1	4.90 a	0.01	
	Site 2	7.38 c	0.01	
	Site 3	9.68 d	0.01	

Site 4	6.87 b	0.01	P<0.05

Different letters indicate significant differences at $p < 0.05$. LSD test was used to compare the parameter concentrations between samples; Ns: non-significant; Min: Minimum; Max: Maximum.
